# Familial Mediterranean Fever in childhood: a single center experience

**DOI:** 10.1186/1546-0096-13-S1-O12

**Published:** 2015-09-28

**Authors:** K Barut, AB Sinoplu, G Yucel, G Pamuk, A Adrovic, S Sahin, O Kasapcopur

**Affiliations:** 1 Istanbul University, Cerrahpasa Medical Faculty, Pediatric Rheumatology, Istanbul, Turkey

## Introduction

Familial Mediterranean fever (FMF) is an autosomal recessively inherited autoinflammatory disease which is clinically manifested with periodic episodes of fever, polyserositis and arthritis. Among the Turkish, Arabic, Armenian and Jewish population the incidence rate ranges between 1 in 500 to 1000. The severity of the disease depends mostly on the MEFV gene mutation variations.

## Objective

The objective of this study is to reveal a single center follow-up experience of a wide amount of childhood FMF patients from different regions around Turkey in terms of the demographic and clinical features, the genetic diversity and treatment response.

## Material-methods

708 children diagnosed with FMF and under treatment of colchicine for at least 6 months that are seen out patiently in our Pediatric Rheumatology Clinic between November 2014 and March 2015 were reviewed retrospectively with the data based on the patient records and also taken from the parents.

## Results

708 patients consisting 362 males and 246 females that are diagnosed with FMF were included in the study. The mean age of the patients by the time of the study was found to be 12,3 ± 4,4 years, while the mean age at the onset of the disease was 4,8 ± 3,4 years and at diagnosis was 7,3 ± 3,8 years. The consanguinity rate was resulted to be %29,2 and positive family history was detected in 370 (52,3%) patients.

In 634 (89,5%) of patients, episodes of abdominal pain for at least 6 hours due to peritonitis; in 629, (88.8%) periods of fever for at least 12 hours and in 122 (17,2%), chest pain probably due to pleuritis was found. 213 (30,1%) patients experienced erysipelas like erythema and 288 (40,7%) were diagnosed with findings of arthritis that last for at least a day. In 467 (66%) cases exertional leg pain and in 29 (4,1%) myalgia are among the complaints and enthesitis was reported in 26 (3,7%) patients. Pericarditis was developed only in 2 (0,3%) patients. The mean duration of the attack was found to be 64,8 ± 38,5 hours. In 38 (53,4%) patients with appendectomy was performed due to unresolved episodes of abdominal pain.

The patients were investigated about the MEFV gene mutations and M694V homozygote mutation was found in 154 (21,8%) and M694V heterozygote in 141 (19,8%) children. All the other mutations in exon 10 region (M680I, V726A, M694I) in a compound heterozygous manner with M694V mutation were detected to be in 90 (12,7%) cases and the rate of patients that is a carrier of only one copy of exon 10 region mutations other than M694V was %13. The rest of the mutations (in exon 2,3,5 regions) were revealed in 45 (6,4%) children. In 45 (6,4%) of patients none of the so far known main mutations were shown.

Amyloidosis had been developed only in two cases both of whom were suffering from the disease process for at least 10 years and showing defective compliance to the colchicine treatment. One of them was a 20 years old M694V homozygote mutation carrier and the other was happened to be compound heterozygous with M694V/M680I mutations.

All of the patients were under colchicine treatment, of which 57 (8,1%) were uncompliant. As the side effects of the treatment were evaluated, diarrhea was found out to be in the first rank with the rate of 47(6.6%), then comes the elevation in serum transaminase levels by 10 (1.4%). Only in one case leukopenia and also in another case alopecia developed.

The response to colchicine treatment was investigated and in 559 (79%) patients no episode occurred in the last year. In 149 (21%) cases periodic episodes were reported by the mean rate of 4,9 ± 4,4 per year (median 3/year, ranging between 1-24/year). After the follow up under treatment for at least 6 months the mean episode duration was decreased to 39,7 ± 29,4 hours (median 24 hours ranging between 6-168 hours).

The patients who are taking their colchicine treatment properly but still complaining about at least 6 episodes of any possible manifestations per year were considered resistant and were found to be in the rate of 47 (6,6%) in our cohort. In resistant cases, the most detected mutations were homozygous M694V (71.4%) in the first range and then compound heterozygosity with M694V/M680I.

## Conclusion

The diagnosis of childhood FMF is frequently encountered in our country. The most severe clinical presentation occurs as a result of M694V and other exon 10 region mutations. With the absolute compliance to treatment; the episodes disappear and amyloidosis, the most dreadful complication of the disease, can be prevented.

